# A critical age: can we reliably measure frailty in critical care?

**DOI:** 10.1186/s13054-017-1704-3

**Published:** 2017-05-31

**Authors:** Richard J. Pugh, Chris M. Thorpe, Christian P. Subbe

**Affiliations:** 1 Department of Anaesthetics, Glan Clwyd Hospital, Bodelwyddan, Denbighshire, Wales LL18 5UJ UK; 2 Department of Anaesthetics, Ysbyty Gwynedd, Bangor, Gwynedd, Wales LL57 2PW UK; 3 Acute, Respiratory and Intensive Care Medicine, Ysbyty Gwynedd, Bangor, Gwynedd, Wales LL57 2PW UK; 4 School of Medical Sciences, Bangor University, Bangor, Gwynedd, Wales LL57 2DG UK

**Keywords:** Frailty assessment, Frailty, Clinical Frailty Scale, Reliability, Critical care, Critically ill

As populations age, the proportion of older patients admitted to critical care units has risen in a number of nations [1]. Age remains an independent predictor of poorer short- and long- term outcomes following critical care admission [1], but the distinction between “physiological” and “chronological” age has long been apparent to critical care clinicians. For the critically ill, assessment of frailty (“a condition characterised by loss of biological reserve and vulnerability to poor resolution of homeostasis following a stressor event” [2]) offers the potential both to inform discussions when escalation of care is being considered, and to identify those who may need a higher level of support in their recovery from critical illness. Although several reports regarding the predictive validity of frailty assessment tools in the critically ill have been published recently [3], study of the reliability of such assessments is virtually absent from the critical care literature.

We therefore undertook a prospective study of consecutive patients admitted to a single UK critical care unit. Frailty was assessed with regards to condition 2 weeks prior to hospital admission using the Clinical Frailty Scale (CFS) [4]. Assessments were performed independently by a medical student and a critical care doctor following interviews with the patient and/or family. Inter-rater reliability was assessed using linear-weighted kappa.

Assessments of frailty were made for 30 patients (median age 70.5 years, 60% male, median Acute Physiology and Chronic Health Evaluation (APACHE) II score 16, median CFS 3 (interquartile range (IQR) 2–5). The frequency distribution of CFS scores is presented in Fig. 1. Linear weighted kappa was 0.64 (95% confidence intervals 0.40 to 0.87; *p* < 0.0001), suggesting a good level of agreement.

**Fig. 1:**
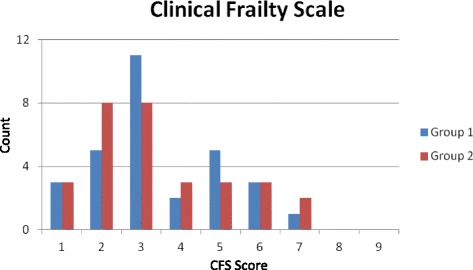
Frequency distribution of Clinical Frailty Scale (*CFS*) scores. Frequency distribution of assessments of CFS (30 patients, each undergoing an assessment by Group 1 (Medical Students) and an assessment by Group 2 (Critical Care doctors))

The major limitations of our preliminary study are that the study population was small, it was single-centre, and that patients with higher CFS scores were relatively under-represented (in line with previous reports [3]). There are potential difficulties in assessing frailty in the context of acute illness [5]; however, in spite of a reliance on proxies (e.g. family members) to make the assessment we have found that with appropriate training the application of frailty assessment tools in the critically ill can be reliable. Given the potential utility of frailty assessment in clinical practice, administration and research, there is a need to evaluate the clinimetric properties of frailty assessment tools in the critically ill with larger, multi-centred studies.

## Abbreviation

CFS: Clinical Frailty Scale
